# Stimulation of soluble guanylate cyclase by vericiguat reduces skeletal muscle atrophy of mice following chemotherapy

**DOI:** 10.3389/fphar.2023.1112123

**Published:** 2023-01-19

**Authors:** Bo-ang Hu, Yu-lin Li, Hai-tao Han, Bin Lu, Xu Jia, Lu Han, Wei-xuan Ma, Ping Zhu, Zhi-hao Wang, Wei Zhang, Ming Zhong, Lei Zhang

**Affiliations:** ^1^ The Key Laboratory of Cardiovascular Remodeling and Function Research, Chinese Ministry of Education, Chinese National Health Commission and Chinese Academy of Medical Sciences, The State and Shandong Province Joint Key Laboratory of Translational Cardiovascular Medicine, Department of Cardiology, Qilu Hospital, Cheeloo College of Medicine, Shandong University, Jinan, Shandong, China; ^2^ Department of General Practice, Qilu Hospital, Cheeloo College of Medicine, Shandong University, Jinan, Shandong, China; ^3^ Department of Geriatric Medicine, Qilu Hospital, Cheeloo College of Medicine, Shandong University, Shandong key Laboratory of Cardiovascular Proteomics, Jinan, Shandong, China

**Keywords:** doxorubicin, soluble guanylate cyclase, skeletal muscle atrophy, protein synthesis, protein degradation, vericiguat

## Abstract

**Background:** The chemotherapeutic doxorubicin (DOX) promotes severe skeletal muscle atrophy, which induces skeletal muscle weakness and fatigue. Soluble guanylate cyclase (sGC) contributes to a variety of pathophysiological processes, but whether it is involved in DOX-induced skeletal muscle atrophy is unclear. The present study aimed to stimulate sGC by vericiguat, a new oral sGC stimulator, to test its role in this process.

**Methods:** Mice were randomly divided into four groups: control group, vericiguat group, DOX group, and DOX + vericiguat group. Exercise capacity was evaluated before the mice were sacrificed. Skeletal muscle atrophy was assessed by histopathological and molecular biological methods. Protein synthesis and degradation were monitored in mice and C2C12 cells.

**Results:** In this study, a significant decrease in exercise capacity and cross-sectional area (CSA) of skeletal muscle fibers was found in mice following DOX treatment. Furthermore, DOX decreased sGC activity in mice and C2C12 cells, and a positive correlation was found between sGC activity and CSA of skeletal muscle fibers in skeletal muscle. DOX treatment also impaired protein synthesis, shown by puromycin detection, and activated ubiquitin-proteasome pathway. Following sGC stimulation, the CSA of muscle fibers was elevated, and exercise capacity was enhanced. Stimulation of sGC also increased protein synthesis and decreased ubiquitin-proteasome pathway. In terms of the underlying mechanisms, AKT/mTOR and FoxO1 pathways were impaired following DOX treatment, and stimulation of sGC restored the blunted pathways.

**Conclusion:** These results unravel sGC stimulation can improve skeletal muscle atrophy and increase the exercise capacity of mice in response to DOX treatment by enhancing protein synthesis and inhibiting protein degradation. Stimulation of sGC may be a potential treatment of DOX-induced skeletal muscle dysfunction.

## Introduction

In 2020, it was estimated that 19 million people were diagnosed with cancer, and more than 9.9 million individuals succumbed to it. Due to this, cancer is still one of the major public health concerns responsible for significant mortality and morbidity worldwide. Anthracyclines are a class of chemotherapeutic agents that are widely used in the treatment of a variety of solid tumor and leukemia cancers, and doxorubicin (DOX) is one of the most common anthracyclines in clinical use (Todorova et al., 2021). However, the accumulation of DOX in skeletal muscle contributes to severe fatigue and muscle loss in cancer patients, which impacts the quality of life (Gilliam and St Clair, 2011). Moreover, a consistent target and a satisfactory and effective therapy for this damage have so far not been achieved.

The major histopathological change of DOX-induced skeletal muscle damage is skeletal muscle atrophy, which is manifested by a reduction in the size of skeletal muscle myocytes (Hiensch et al., 2020; Powers et al., 2020). Meanwhile, it is essential to note that skeletal muscle atrophy is an independent risk factor of worsened cancer prognosis and associated with a higher risk of morbidity and mortality in cancer patients (Barret et al., 2014; Miyamoto et al., 2015). Thus, the molecular pathways related to DOX-induced skeletal muscle atrophy are crucial. The reason for skeletal muscle myocyte atrophy is a result of increased protein degradation as well as decreased protein synthesis. However, the mechanisms underlying these processes remain unclear.

Soluble guanylate cyclase (sGC), which is a nitric oxide (NO)-responsive enzyme, generates the secondary messenger cyclic guanosine monophosphate (cGMP) in eukaryotes (Arnold et al., 1977). cGMP in turn activates downstream effector to regulate a variety of physiological responses (Steinert et al., 2010; Bohlen, 2015). sGC dysfunction leads to the pathogenesis of vasoconstriction, platelet aggregation, inflammation, and apoptosis (Ghofrani et al., 2017; Liu et al., 2020). For skeletal muscle, sGC stimulation can increase the oxygen transport and improve exercise efficiency. In addition, sGC can also modulate muscle microtubule organization and mitochondrial oxidative phosphorylation ultimately improving muscle fatigue (Krishnan et al., 2021). A recent research reported sGC as a potential therapeutic target in DOX-induced heart failure (Todorova et al., 2021). However, whether the sGC is dysfunctional in DOX-induced skeletal muscle atrophy and its role remains to be elucidated. As a new sGC agonist, vericiguat has a dual mode of action. It can not only increase the sensitivity of sGC to endogenous NO, but also directly stimulate sGC to increase the formation of cGMP (Campbell et al., 2022). In this study, we investigated the effects of vericiguat on skeletal muscle atrophy in DOX mice, and discussed the mechanism of sGC stimulation improving skeletal muscle atrophy.

## Materials and methods

### Animals and experimental protocol

All animal procedures were performed in accordance with animal protocols approved by Shandong University Institutional Animal Care and Use Committee. Eight-week-old male C57BL/6J mice were used for this study. They were housed in standard cages under a 12 h light-dark cycle at 22 ± 1°C and 60 ± 5% humidity and received a chow diet and water *ad libitum*. The doxorubicin-induced skeletal muscle atrophy model was performed as described in Gilliam et al. (2016). Mice received an intraperitoneal injection of DOX (MCE HY-15142) dose of 20 mg/kg and control animals received an equal volume of saline. To test the therapeutic potential of vericiguat, mice were randomly divided into four groups (seven mice per group): control group, vericiguat group, DOX group, and DOX + vericiguat group. Mice in the vericiguat-treated group received a gavage of vericiguat (MCE HY-16774) for two weeks prior to injection of DOX and continued until 72 h after the DOX injection. The dosage of vericiguat was set to 3 mg/kg/day, as used previously in other studies (Follmann et al., 2017; Cai et al., 2022). Mice were monitored daily and then euthanized 72 h post-injection. Mice were weighed pre and post-injection for calculation of injection volume and changes in the body weight. Skeletal muscle tissues were dissected out, removed, weighed, and snap-frozen in liquid nitrogen prior to storage at −80°C for future analysis. Whole blood was drawn and centrifuged at 3000 g for 15 min at 4°C. Serum was removed and kept frozen at −80°C for later determination.

### Cell culture

Mouse skeletal muscle cell line C2C12 was cultured in high glucose DMEM (Gibco) supplemented with 10% FBS (Gibco) at 37°C in a humid environment containing 5% CO_2_. C2C12 myoblasts were induced differently from muscle tubes for 7 days in a medium containing high glucose DMEM and 2% horse serum (Gibco), refreshing media every 24 h. C2C12 myotube cells were treated with different concentrations of DOX, while control cells were treated with DMSO of the same volume as the DOX solution for 24 h. After 24 h, we subjected the myotube cells to several assays.

### Histological analysis

Mouse tibialis anterior, gastrocnemius, and soleus muscle were fixed in 4% paraformaldehyde, then bisected at the mid-belly and embedded in paraffin. The hematoxylin and eosin (HE) was used to stain the 5 μm sections from the muscle center. Images were acquired using an Olympus DP72 digital imaging system (Olympus Corporation), and the CSA of muscle fibers was calculated using Image-Pro Plus.

### Western blot analysis

Proteins were extracted from tissues and cells with RIPA buffer (Sparkjade, EA0002) containing protease and phosphatase inhibitors, added to each well (30ug/well), fractionated by SDS-PAGE, and transferred to PVDF membranes (Millipore Ipv304F0). The membranes were blocked in 5% non-fat dried milk/Tween 20-tris buffered saline (TBST) for 1 h and incubated overnight at 4°C with primary antibodies: Anti-Fbx32 (Abcam ab168372), Anti-MuRF1 (Proteintech 55456-1-AP), Anti-Gapdh (Proteintech 66004-1-I), Anti-GUCY1A3 (Proteintech 12605-1-AP), Anti-GUCY1B3 (Abcam ab154841), Anti-AKT (CST 4685S), Anti-p-AKT (CST 4060S), Anti-mTOR (Proteintech 66888-1-IG), Anti-p-mTOR (CST D9C2), Anti-p70S6K (CST E8K6T), Anti-p-p70S6K (CST D5U1O), Anti-4EBP1 (CST 53H11), Anti-p-4EBP1 (CST 236B4). After cleaning three cycles with TBST, membranes were incubated with appropriate horseradish peroxidase-conjugated secondary antibodies at a 1:5000 dilution, then washed three times in TBST and observed by enhanced chemiluminescence. ImageJ software was used for analysis. Gapdh protein levels were determined as housekeeping control for normalization. All experiments were performed at least three times.

### Puromycin assay

Previous studies have found that minimal amounts of puromycin incorporation in neosynthesized proteins directly reflect the translation rate of mRNA (Schmidt et al., 2009). For the *in vitro* study, 1 μM puromycin (BioFroxx 1299MG025) was added to the medium 30min before C2C12 was collected. For the *in vivo* study, mice were injected intraperitoneally with 0.040 μmol/g body weight of puromycin 30min before euthanasia. Then, muscle tissues were collected in the order of injection. We used the anti-puromycin antibody (Sigma MABE343) by western blot to detect in the samples, in order to quantify the protein synthesis rate and reflect the protein synthesis (Hain et al., 2021).

### Hanging grid test

Mice were individually placed at the center of a grid (2 mm wire thickness). The screen was held 50 cm above a pad. The grid was inverted upside down with the head declining first. The duration of hanging was recorded in three independent trials conducted at least 30 min apart (Jiang et al., 2020; Shang et al., 2020). The data of all three trials were averaged.

### Forelimb grip strength test

The forelimb grip strength test was performed using a tension strength meter (Handpi HP-5N). Mice were held by the tail, and they grasped the horizontal bar that was attached to the dynamometer with their fore paws. They were then gently pulled by the tail until they released their grip with their paws. Each mouse was measured three times and took the average.

### Running tolerance test

After an acclimation period, non-treated mice were tested for maximal exercise capacity on a treadmill and reassessed 72 h after DOX injection. The running was started at 10 m/min with a slope angle of 0°, and then the speed and slope angle were increased by 3 m/min and 2° every 3 min until they reached 29 m/min and 14°, respectively (Caru et al., 2019; Jiang et al., 2020). When the mouse being tested did not return to the track for >10 s and concomitantly exhibited a markedly diminished response to external stimuli (Electrical stimulation), the mouse was moved out from the treadmill, and the exhaustive running time and distance were recorded.

### Determination of cGMP

Skeletal muscle samples were rapidly frozen in liquid nitrogen after different treatments, according to the requirements of the cGMP Elisa kit (CST 8020). 1mL of lysis buffer (CST 9803) was added to 30 mg of muscle tissue. After homogenization, the supernatant was collected by centrifugation at 4°C (14000rpm, 10 min). For cell measurement, C2C12 cells were seeded in 96-well plates and received different interventions after differentiation. The cells were rinsed in ice-cold PBS and then added 100 ul/well lysis buffer for 5 min on ice. cGMP within the sample competed with a fixed amount of horseradish peroxidase-labeled cGMP for sites on the monoclonal antibody and was quantified by measurement of relative light unit (RLU) at 425 nM.

### Serum testing

Serum was analyzed for glucose, insulin, and CK using colorimetry kits (Jiancheng Biology Engineering Institute, Nanjing, China), according to the manufacturer’s protocol.

### Statistical analysis

Statistical analysis was performed using SPSS 19.0. All data were expressed as mean ± SD. The Shapiro-Wilk test was used to verify the normal distribution of the data, and then the homogeneity of variance of the data was tested. Unpaired Student’s test was used for comparisons between two groups and two-way ANOVA test was used for comparisons among multiple groups. When a significant main effect was observed, multiple comparisons testing was completed using Bonferroni tests. Correlation between cGMP concentration and skeletal muscle fiber size was evaluated using Pearson’s correlation coefficient. *p*-value < 0.05 was considered statistically significant.

## Results

### Muscle atrophy caused by DOX is related to the decrease of sGC activity

**FIGURE 1:**
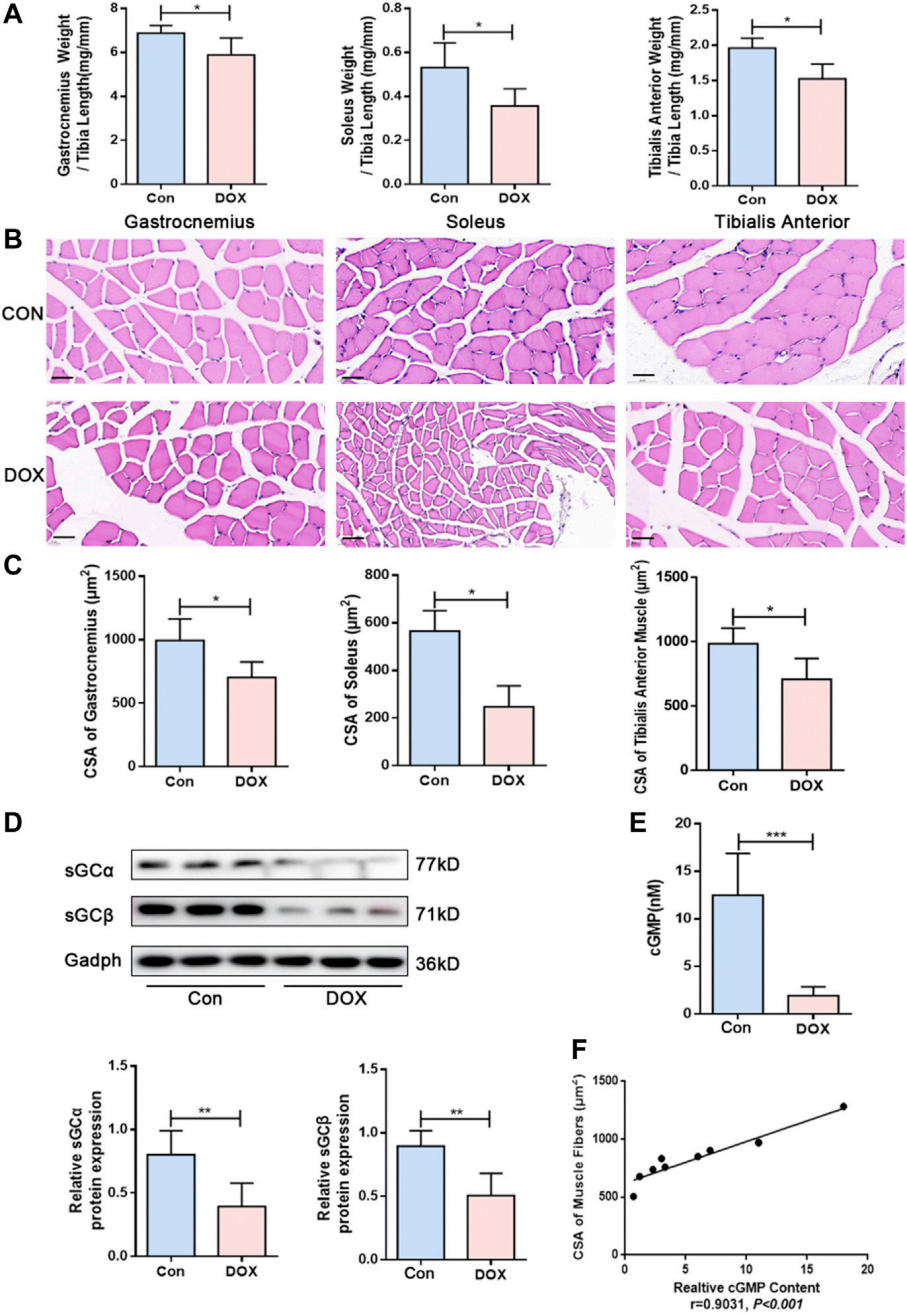
DOX induced skeletal muscle atrophy and decreased sGC activity in mice. **(A)** The ratios of the gastrocnemius muscle, soleus muscle, and tibialis anterior muscle weight to tibia length (mg/mm). **(B)** Representative HE staining of different muscle CSA (Scale bar: 50 μm). **(C)** CSA of muscle fibers (μm^2^). **(D)** Expressions of sGCα and sGCβ detected through western blot. **(E)** Detection of cGMP content in gastrocnemius muscle by Elisa. **(F)** Correlation between CSA of gastrocnemius muscle fibers and cGMP content. n = 5; *p < 0.05, **p < 0.01, ***p < 0.001.

C57BL/6J mice treated with DOX (20 mg/kg, intraperitoneally) showed reduced skeletal muscle mass and loss of CSA, as previously reported (Hiensch et al., 2020). The ratio of skeletal muscle weight to tibial length is an important index to evaluate skeletal muscle quality. Compared with the control group, the ratios of the gastrocnemius muscle, soleus muscle, and tibialis anterior muscle weight to tibial length were decreased in the DOX group (*p* < 0.05 for all) (Figure 1A). The results of HE staining showed that DOX treatment exhibited apparent variations in size and arrangement in skeletal muscle fibers (Figure 1B). The CSA of skeletal muscle fibers of gastrocnemius muscle, soleus muscle, and tibialis anterior muscle in the DOX group were significantly decreased compared with the control group (*p* < 0.05 for all) (Figure 1C).

sGC is a heterodimer composed of two homologous subunits α and β. The most commonly studied isoform is α1β1 protein, which is present in skeletal muscle (Feussner et al., 2001; Derbyshire and Marletta, 2012). According to western blot analysis results, sGCα and sGCβ expressions presented a significant decrease in gastrocnemius muscle following DOX treatment (*p* < 0.01 and *p* < 0.01) (Figure 1D). The content of cGMP in tissue can reflect the activation of sGC (Hall et al., 2019), and cGMP production is triggered by stimulation of sGC (Friebe et al., 2020). We also observed that muscle cGMP levels were downregulated in the DOX group compared with the control group (*p* < 0.001) (Figure 1E). Moreover, Pearson’s correlation analysis was used to evaluate the correlation between cGMP content and skeletal muscle fiber size. The results showed a positive correlation between CSA of skeletal muscle fibers and cGMP content in skeletal muscle (r = 0.9031, *p* < 0.001) (Figure 1F).

### Vericiguat alleviated muscle atrophy and muscle injury caused by DOX

**FIGURE 2:**
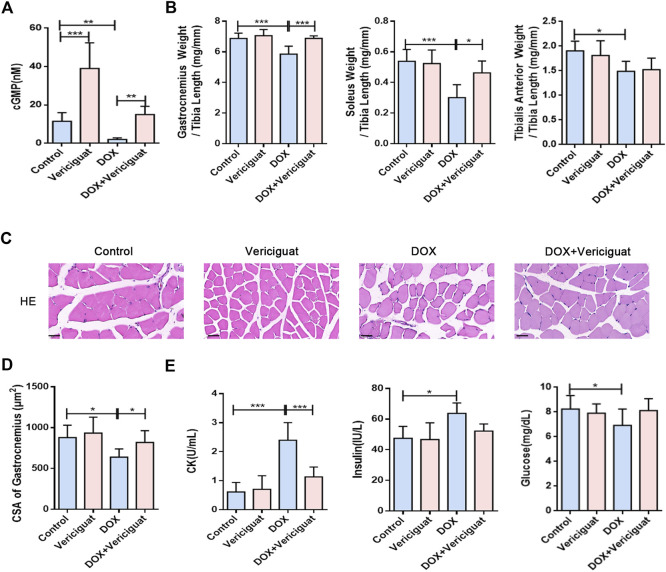
Vericiguat alleviated skeletal muscle atrophy and muscle injury. **(A)** Detection of cGMP content in mice by Elisa. **(B)** The ratios of the gastrocnemius muscle, soleus muscle, and tibialis anterior muscle weight to tibia length (mg/mm). **(C)** Representative HE staining of gastrocnemius muscle CSA (Scale bar: 50 μm). **(D)** CSA of gastrocnemius muscle fibres in different treatment (μm^2^). **(E)** Serum levels of CK, insulin and glucose. n = 5–7; *p < 0.05, **p < 0.01, ***p < 0.001.

The NO-sGC-cGMP pathway plays a vital role in skeletal muscle (muscle fiber type, fatigability and postexercise force recovery partly) (Moon et al., 2017) and muscle microvessels (vasodilation to improve perfusion) (Genders et al., 2011; Nyberg et al., 2015). As a stimulator of sGC, vericiguat can not only stimulate sGC directly but also cooperate with NO, resulting in the increase of cGMP (Kang and Lamb, 2022). Previous studies have shown that vericiguat of 3 mg/kg can significantly increase the content of cGMP in mice (Cai et al., 2022). Our results show compared with the control group, the content of cGMP was decreased in the DOX group (*p* < 0.05). After vericiguat treatment, the content of cGMP in mice was increased accordingly (Figure 2A).

Next, we examined the effects of vericiguat on skeletal muscle mass and skeletal muscle CSA (mainly gastrocnemius) in DOX mice. Compared with DOX group, the ratios of gastrocnemius muscle and soleus muscle to tibia length were increased (*p* < 0.001 and *p* < 0.05), and the ratio of tibialis anterior muscle to tibia length was increased non-significantly in the DOX + vericiguat group (*p* > 0.05) (Figure 2B). HE staining revealed that stimulation of sGC could recover the loss of CSA in gastrocnemius muscle and marked variations in skeletal muscle arrangement induced by DOX as showed in the DOX + vericiguat group (Figure 2C). Moreover, muscle fiber CSA was significantly higher in the DOX + vericiguat group than in the DOX group (*p* < 0.05) (Figure 2D).

We assessed skeletal muscle injury by testing four groups of serum parameters. DOX groups showed substantial increased levels of CK and insulin and decreased levels of glucose and in serum (*p* < 0.001, *p* < 0.05, and *p* < 0.05, respectively) (Figure 2E). Vericiguat could improve the increase of CK induced by DOX, but had no effect on blood glucose and insulin in DOX mice.

### DOX downregulated sGC activity, decreased protein synthesis and increased protein degradation in C2C12 myotubes

**FIGURE 3:**
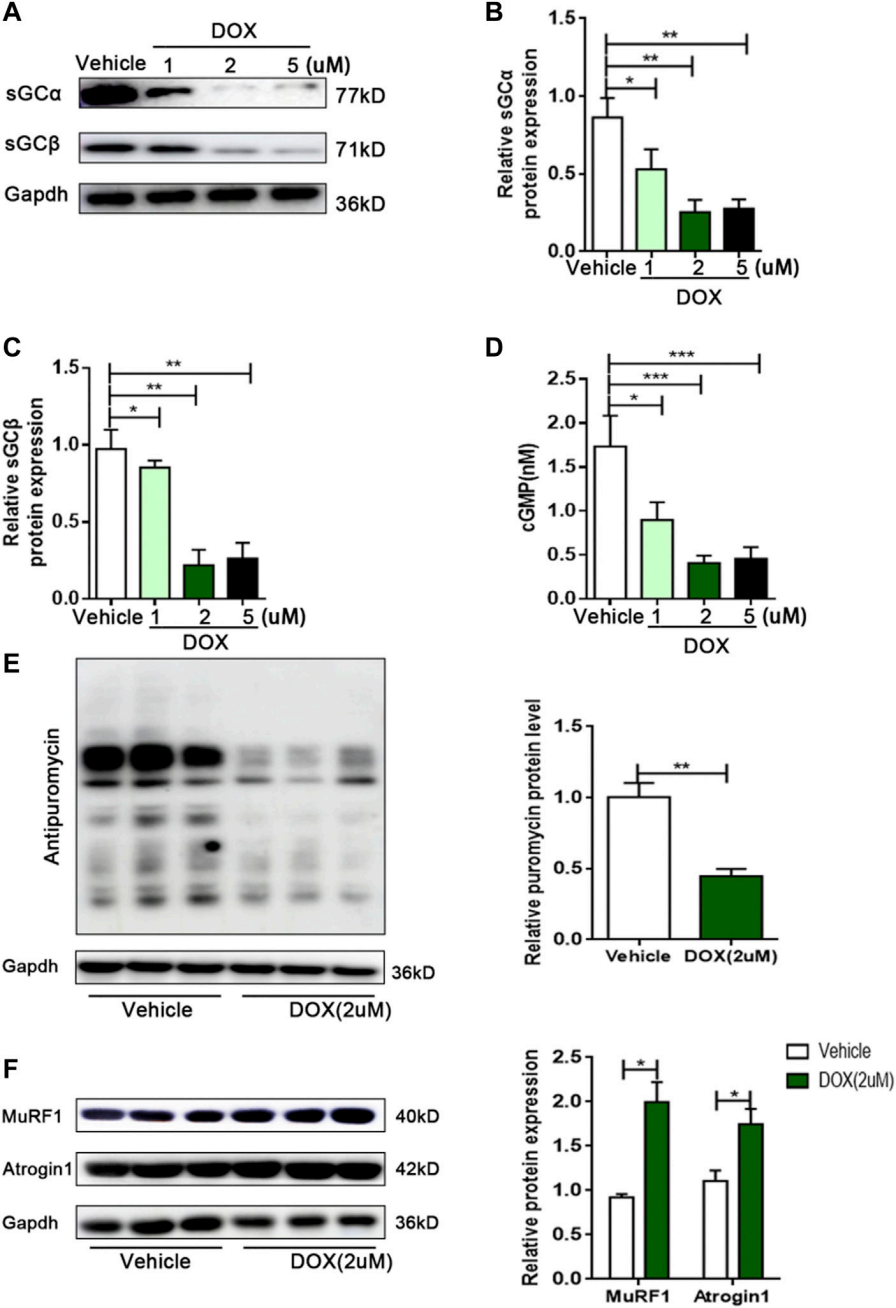
DOX leads to decreased sGC activity, decreased protein synthesis, and increased protein degradation in C2C12 myotubes. **(A)** Representative of western blot images of sGCα and sGCβ expressions in C2C12 cells treated with the vehicle DMSO or 1, 2 and 5 μM DOX for 24 h. **(B)** Quantification of the relative levels of sGCα proteins from panel **(A)**. **(C)** Quantification of the relative levels of sGCβ proteins from panel **(A)**. **(D)** Detection of cGMP content in C2C12 myotubes by Elisa. **(E)** Representative immunoblots of puromycin incorporation in C2C12 myotubes treated with 2 μM DOX. **(F)** Representative immunoblots of MuRF1 and Atrogin1 in C2C12 myotubes treated with 2 μM DOX. n = 3; *p < 0.05, **p < 0.01, ***p < 0.001.

Although DOX reduced the sGC/cGMP pathway *in vivo*, it is unclear whether DOX can also exert this effect in skeletal muscle cells. Next, we investigated the effect of DOX on sGC expression in C2C12 myotubes. Compared with vehicle, the expressions of sGCα and sGCβ in C2C12 myotubes were significantly reduced by 2 μm of DOX treatment for 24 h (*p* < 0.01 for both) (Figures 3A–C). In addition, the content of cGMP in C2C12 myotubes treated with DOX was significantly decreased than vehicle group (*p* < 0.001) (Figure 3D). Muscle atrophy can result from increased protein degradation and decreased protein synthesis (Vaughan et al., 2013). In the DOX group, the amount of puromycin infiltration was significantly reduced (*p* < 0.01) (Figure 3E). At the same time, DOX treatment could significantly increase the expressions of MuRF1 and Atrogin1, which are important regulators of ubiquitin-mediated protein degradation in skeletal muscle (*p* < 0.05 for both) (Figure 3F). These results suggested that DOX impaired sGC activity, protein synthesis, and increased degradation in skeletal muscle cells.

### Vericiguat treatment increased protein synthesis and decreased protein degradation in DOX mice

**FIGURE 4:**
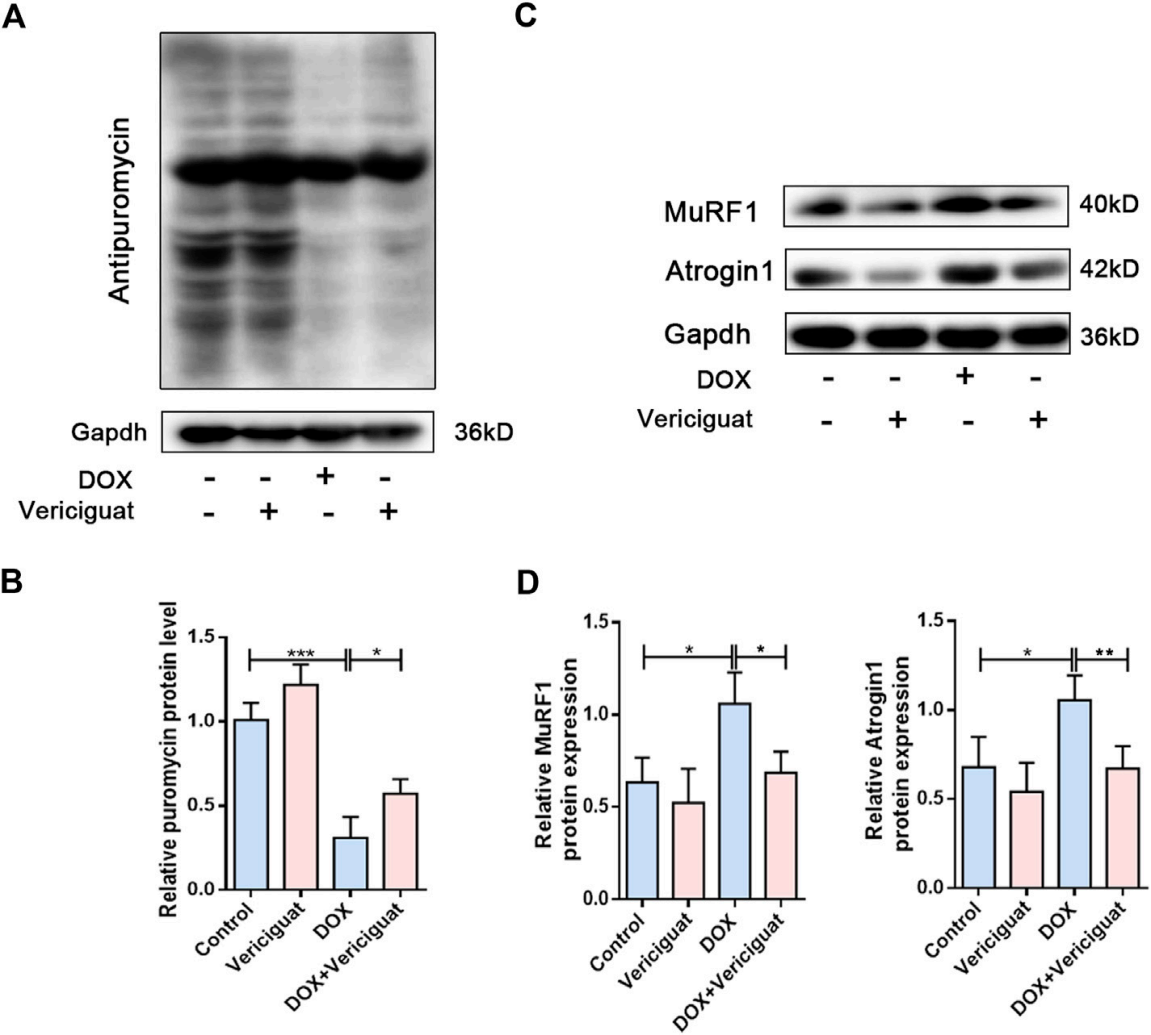
Vericiguat increased protein synthesis. **(A)** Expression of puromycin detected through western blot. **(B)** Relative protein expression levels of puromycin. **(C)** Expression of MuRF1 and Atrogin1 detected through western blot. **(D)** Relative protein expression levels of MuRF1 and Atrogin1. n = 5; *p < 0.05, **p < 0.01, ***p < 0.001.

Next, we detected protein degradation and protein synthesis in DOX mice. Consistent with expected results, the protein synthesis of DOX mice was significantly decreased than control group (*p* < 0.001). Next, vericiguat could restore partial protein synthesis in DOX mice (*p* < 0.05) (Figures 4A,B). We also found DOX increased the expressions of muscle atrophy markers MuRF1 and Atrogin1, and vericiguat decreased MuRF1 and Atrogin1 expressions (*p* < 0.05) (Figures 4C,D). These findings indicated that vericiguat treatment increased protein synthesis and decreased protein degradation in DOX mice.

### Vericiguat restored AKT/mTOR and FoxO1 pathways in DOX mice

**FIGURE 5:**
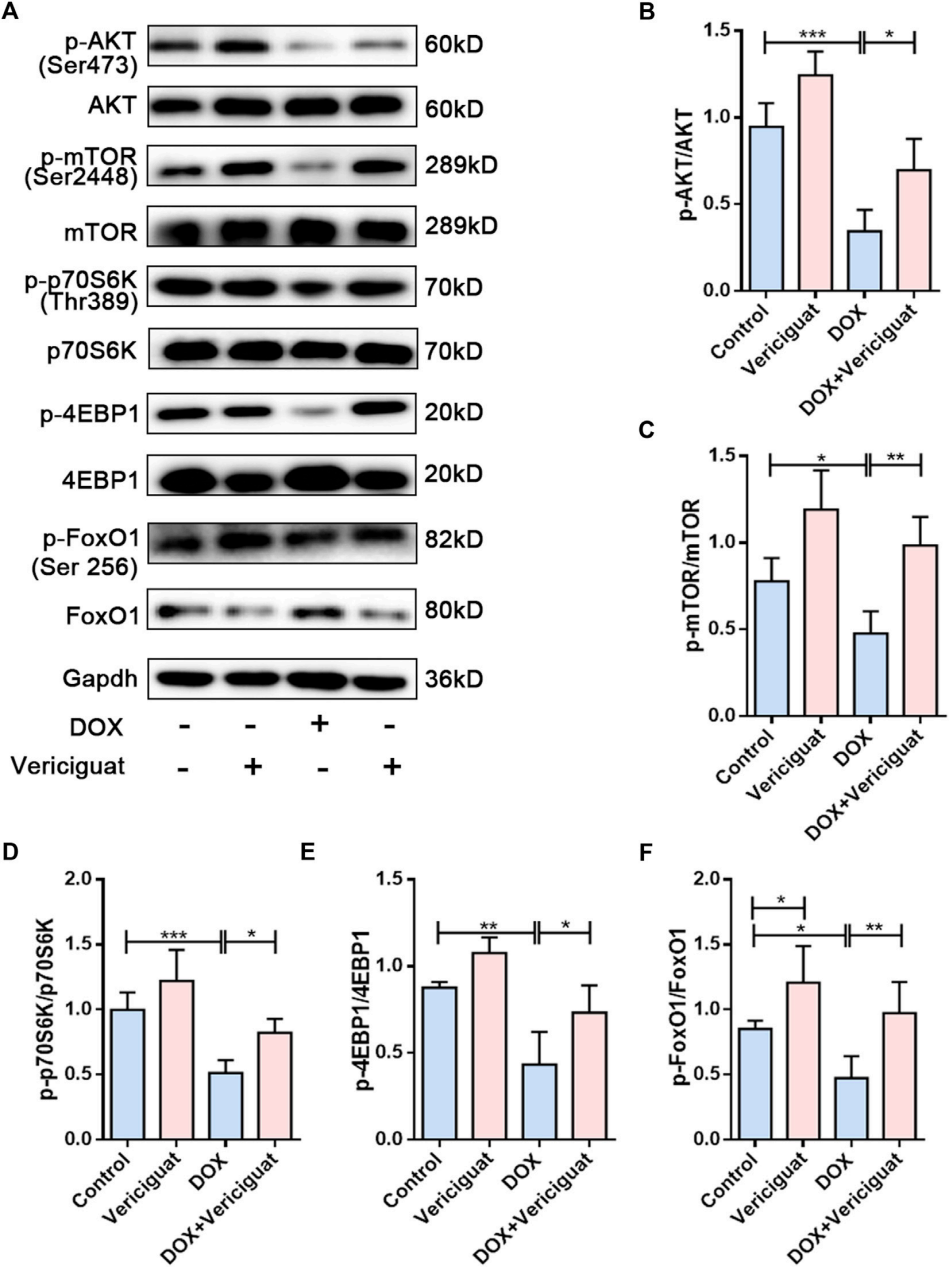
Vericiguat restored AKT/mTOR and FoxO1 pathways in DOX mice. **(A)** Expression of p-AKT, AKT, p-mTOR, mTOR, p-p70S6K, p70S6K, p-4EBP1, 4EBP1, p-FoxO1 and FoxO1 detected through western blot. **(B–F)** Relative protein expression levels of p-AKT, AKT, p-mTOR, mTOR, p-p70S6K, p70S6K, p-4EBP1, 4EBP1, p-FoxO1 and FoxO1. n = 5; *p < 0.05, **p < 0.01, ***p < 0.001.

To further investigate the molecular mechanism by which vericiguat improves muscle atrophy. We examined AKT/mTOR, a key signaling pathway for anabolic. DOX resulted in decreased phosphorylation levels of AKT, mTOR, and its downstream signaling targets p70S6K and 4EBP1. Furthermore, the phosphorylation levels of AKT, mTOR, p70S6K and 4EBP1 were improved after vericiguat treatment at 3 mg/kg daily (*p* < 0.05) (Figures 5A–E). Taken together, these results indicated that vericiguat could rescue DOX-induced protein synthesis reduction by activating the AKT/mTOR pathway.

Activation of the forkhead transcription factor family is involved in the activation of proteolytic pathways in skeletal muscle by increasing the transcription of MuRF-1 and Atrogin1. Next, we investigated the expression of FoxO1. We found decreased FoxO1 phosphorylation and increased total FoxO1 expression in the muscles of the DOX group compared with the muscles of the control group (*p* < 0.05). Vericiguat treatment was able to increase the phosphorylation of FoxO1 and decrease FoxO1 expression (*p* < 0.05) (Figures 5A,F). These results suggested vericiguat might decrease protein degradation by inhibiting FoxO1 pathway.

### Vericiguat improved the exercise ability of DOX mice

**FIGURE 6:**
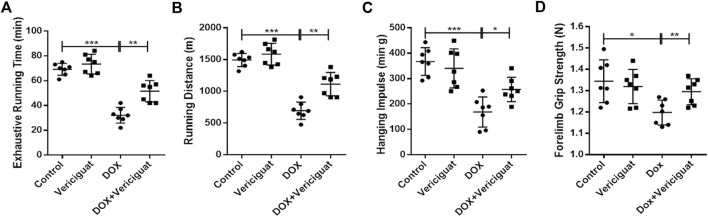
Veirciguat improved the decrease of exercise ability by DOX in mice. **(A)** Exhaustive running time (min). **(B)** Running distance (m) **(C)** Hanging impulse (min^.^g). **(D)** Forelimb grip strength (N). n = 7; *p < 0.05, **p < 0.01, ***p < 0.001.

Finally, we evaluated the recovery effect of vericiguat on exercise capacity in DOX mice. The results showed that the exhausting running time, running distance, grid-hanging time, and forelimb grip strength were significantly lower in the DOX group than in the control group. Compared with the DOX group, the exhausting running time, running distance, grid-hanging time, and forelimb grip strength in the DOX + vericiguat group were significantly ameliorated (*p* < 0.01, *p* < 0.01, *p* < 0.05 and *p* < 0.01, respectively) (Figures 6A–D). Although stimulation of sGC did not increase the exercise capacity of normal mice, these findings suggested that it protected mice from the decline of exercise capacity induced by DOX.

## Discussion

In our present study, we found that skeletal muscle atrophy was present in mice in response to DOX treatment. One of the reasons for these changes could be the decreased activity of sGC, which resulted in the inhibition of protein synthesis and activation of protein degradation. By restoring protein synthesis through the AKT/mTOR pathway and inhibiting protein degradation by FoxO1 pathway, stimulation of sGC alleviated the skeletal muscle atrophy and enhanced the exercise capacity of mice following DOX treatment.

Skeletal muscle atrophy is the main pathological characteristic of DOX-induced skeletal muscle dysfunction (Hulmi et al., 2018). In this study, we found that the CSA of skeletal muscle was decreased in DOX mice. Meanwhile, we recorded DOX significantly decreased the ratio of skeletal muscle weight (tibialis anterior muscle, gastrocnemius muscle, and soleus muscle) to tibial length. Intriguingly, we observed that DOX decreased sGC activity in mice and C2C12 cells, and a positive correlation was found between sGC activity and CSA of skeletal muscle fibers in skeletal muscle, which indicated potential involvement of sGC activity in the development and progression of DOX-induced skeletal muscle atrophy. Our study further showed that stimulation of sGC could improve the CSA of skeletal muscle, reduce serum CK level and enhance the ratio of skeletal muscle weight (gastrocnemius muscle and soleus muscle) to tibial length. DOX promotes rapid skeletal muscle damage, leading to muscle weakness and fatigue in patients (Sorensen et al., 2016). This DOX-induced muscle weakness and fatigue are related to decreased ability to perform activities of daily living (Miyamoto et al., 2015). In our study, we found a clear decline of exhausting running time, running distance, grid-hanging time, forelimb grip strength, and elevated CK level in mice following DOX treatment. These results indicated skeletal muscle damage and decreased muscle strength in DOX mice. Stimulation of sGC could improve the exhausting running time, running distance, grid-hanging time, forelimb grip strength, and CK level. Therefore, stimulation of sGC, which restored skeletal muscle atrophy, could improve the motor ability of DOX mice.

The reason for skeletal muscle atrophy is a result of increased protein degradation as well as decreased protein synthesis. Enhanced protein degradation occurs mostly through activation of the ubiquitin-proteasome pathway (Haberecht-Muller et al., 2021). Atrogin-1 and MuRF-1 control polyubiquitination, which is a rate-limiting step in the ubiquitin‐proteasome proteolysis pathway. In our study, we found Atrogin-1 and MuRF-1 were markedly elevated in mice and C2C12 cells following DOX treatment, which agreed with previous studies (Kavazis et al., 2014). Our study further showed that stimulation of sGC could decrease Atrogin-1 and MuRF-1. The mechanism that stimulation of sGC ameliorated ubiquitin-proteasome pathway might be *via* restoring the FoxO1 pathway. FoxO1, which is an up-regulator of Atrogin-1 and MuRF-1, plays an important role in regulating protein ubiquitination (Das et al., 2022). Therefore, we explored the changes in the FoxO1 pathway. In our experiments, DOX could activate FoxO1 pathway. These results indicated DOX could increase the ubiquitin-proteasome pathway by FoxO1 pathway. Stimulation of sGC decreased the ubiquitin-proteasome pathway and restored FoxO1 pathway. Therefore, stimulation of sGC might decrease the ubiquitin-proteasome pathway by FoxO1 pathway.

In addition to enhancing protein degradation, DOX could also decrease protein synthesis. Muscle protein synthesis was reported to diminish after 20 h following doxorubicin treatment (Nissinen et al., 2016). In our present study, we observed that the protein synthesis was decreased in mice and C2C12 cells following DOX treatment through puromycin detection. Meanwhile, AKT/mTOR pathway is the main driver of muscle protein synthesis (Jaiswal et al., 2022). Not surprisingly, we found that DOX reduced the protein expression levels of p-AKT and p-mTOR. These results revealed that DOX could reduce protein synthesis by AKT/mTOR pathway. Notably, stimulation of sGC increased protein synthesis and restored the blunted AKT/mTOR pathway. These results indicate that stimulation of sGC might increase skeletal muscle protein synthesis by enhancing AKT/mTOR pathway.

## Conclusion

Our results suggest that impaired sGC can aggravate skeletal muscle atrophy and decrease motor ability of mice following DOX treatment. By enhancing protein synthesis and inhibiting protein degradation, stimulation of sGC can improve skeletal muscle atrophy and thereby increase the exercise capacity of mice in response to DOX treatment. Our findings thus identify that stimulation of sGC may be a potential treatment of DOX-induced skeletal muscle dysfunction.

## Data Availability

The original contributions presented in the study are included in the article/supplementary material, further inquiries can be directed to the corresponding authors.
